# The role of pyroptosis and gasdermin family in tumor progression and immune microenvironment

**DOI:** 10.1186/s40164-023-00464-5

**Published:** 2023-12-08

**Authors:** Mengyuan Li, Ping Jiang, Yuhan Yang, Liting Xiong, Shuhua Wei, Junjie Wang, Chunxiao Li

**Affiliations:** Department of Radiation Oncology, Peking University Third Hospital, Beijing, 100191 China

**Keywords:** Pyroptosis, Gasdermin family, Cell death, Tumor progression, Immunotherapy, Tumor microenvironment

## Abstract

Pyroptosis, an inflammatory programmed cell death, distinguishes itself from apoptosis and necroptosis and has drawn increasing attention. Recent studies have revealed a correlation between the expression levels of many pyroptosis-related genes and both tumorigenesis and progression. Despite advancements in cancer treatments such as surgery, radiotherapy, chemotherapy, and immunotherapy, the persistent hallmark of cancer enables malignant cells to elude cell death and develop resistance to therapy. Recent findings indicate that pyroptosis can overcome apoptosis resistance amplify treatment-induced tumor cell death. Moreover, pyroptosis triggers antitumor immunity by releasing pro-inflammatory cytokines, augmenting macrophage phagocytosis, and activating cytotoxic T cells and natural killer cells. Additionally, it transforms “cold” tumors into “hot” tumors, thereby enhancing the antitumor effects of various treatments. Consequently, pyroptosis is intricately linked to tumor development and holds promise as an effective strategy for boosting therapeutic efficacy. As the principal executive protein of pyroptosis, the gasdermin family plays a pivotal role in influencing pyroptosis-associated outcomes in tumors and can serve as a regulatory target. This review provides a comprehensive summary of the relationship between pyroptosis and gasdermin family members, discusses their roles in tumor progression and the tumor immune microenvironment, and analyses the underlying therapeutic strategies for tumor treatment based on pyroptotic cell death.

## Background

Pyroptosis, a form of programmed inflammatory cell death, was initially identified by Zychlinsky in 1992, observing this unique cell death in macrophages infected with *Shigella flexneri* [1]*.* Further investigations unveiled that the activation of caspase-1 during *Shigella flexneri-*induced macrophage death resulted in the secretion of mature IL-1β [2]. In 2001, Cookson et al. demonstrated that this novel form of death significantly differs from apoptosis, characterized by cell swelling and lysis rather than cell shrinkage and intact membranes [3]. Consequently, this distinct form of programmed inflammatory cell death is termed pyroptosis [3, 4].

**Fig. 1:**
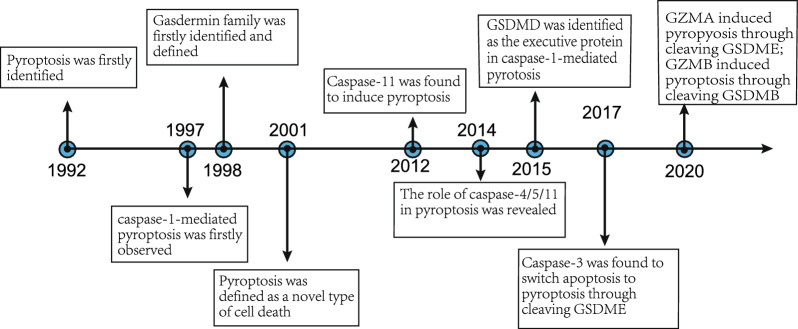
Time course study of pyroptosis

Pyroptosis is intricately linked to various processes, such as membrane pore formation, cell swelling, cell membrane rupture, and release of cellular contents, including pro-inflammatory factors such as interleukin-1β (IL-1β) and IL-18 [5–7]. In addition, researchers have identified that non-canonical caspase-11 can induce cell death independently of caspase-1 [8]. Subsequent studies have elucidated the mechanism by which caspase-11 executes pyroptosis, involving the cleavage of gasdermin D (GSDMD) in response to stimulation by Gram-negative bacteria [9] (Fig. 1).

In 2015, GSDMD emerged as a pivotal executive protein involved in caspase-1-mediated pyroptosis. Upon activation, caspase-1 cleaves GSDMD into GSDMD-N and GSDMD-C, precipitating the release of IL-1β and IL-18 and ultimately leading to pyroptosis [10]. Belonging to the gasdermin (GSDM) family and is accompanied by other members such as GSDMA, GSDMB, GSDMC, GSDME (also known as DFNA5), and pejvakin (PJVK) (also known as DFNB59) [11]. Although subsequent studies have elucidated the involvement of GSDMA and other GSDMs in tumor progression and pyroptosis [12–15], the precise connection between pyroptosis, the GSDM family, and tumors remains unclear.

Recently, Zhang et al. demonstrated that GSDME can induce pyroptosis in tumor cells, thereby activating anti-tumor immunity by enhancing T cell-mediated responses [16]. In the realm of tumor immunotherapy, chimeric antigen receptor T cell (CAR-T) therapy has achieved remarkable success, particularly in hematological malignancies [17, 18]. Notably, researchers found that CAR-T therapy can also stimulate pyroptosis through the release of granzyme B (GZMB). However, it is important to acknowledge that the inflammatory factors induced by pyroptosis may contribute to cytokine release syndrome (CRS), a common and severe complication associated with CAR-T therapy, which limits its broader clinical application [19]. These mechanisms hold promise for the enhancement antitumor immunity.

Consequently, the intricate interplay among pyroptosis, the GSDM family, and tumors requires in-depth exploration. Despite some strides in understanding pyroptosis and the GSDM family, the precise mechanisms and their implications for malignant tumors remain inadequately elucidated. Therefore, additional research is imperative to delve into the roles of pyroptosis and the GSDM family in cancer progression and the tumor microenvironment, and such investigations will contribute to the development of more effective strategies for antitumor treatment.

## The characteristics of pyroptosis distinguished from other forms of cell death

Pyroptosis, a form of inflammatory programmed cell death, shares certain features with apoptosis and exhibits notable distinctions. Both pyroptosis and apoptosis involve DNA damage, chromatin condensation, and dependence on caspases [21–24]. However, pyroptosis cells manifest distinctive bubble-like protrusions on the cell membrane surface and undergo cell swelling before membrane rupture, setting them apart from apoptotic cells [25]. Although membrane blebbing occurs in both pyroptosis and apoptosis, the morphological characteristics of pyroptosis remain uniquely identifiable [26, 27]. Moreover, pyroptosis is characterized by cell swelling, lysis, and the release of pro-inflammatory factors such as IL-1β, IL-18, and high-mobility group box protein 1 (HMGB1) [28–30]. This release of pro-inflammatory factors in pyroptosis triggers inflammation, distinguishing it from apoptosis, which preserves cell membrane integrity and does not induce an inflammatory response [31]. Therefore, although pyroptosis and apoptosis share some commonalities in the initial cellular events, their distinct morphological features and the inflammatory nature of pyroptosis underscore their unique roles in programmed cell death pathways.

**Fig. 2:**
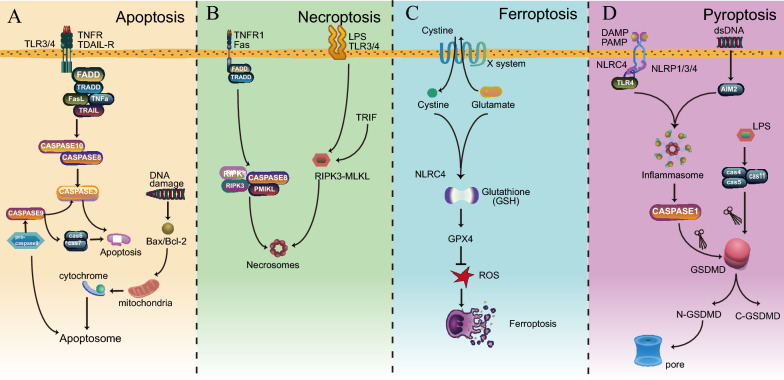
Characteristics of different forms of cell death. **A** In the apoptosis pathway, several death receptors, such as Fas, TNFR, TRAIL-R, and TLRs, recruit FADD or TRADD to activate caspase-8 and -10. Activated caspase-8/10 facilitates the activation of caspase-3, leading to the induction of apoptosis. Additionally, DNA damage can activate BCL family proteins, facilitating the release of cytochrome C into the cytoplasm, which further induces apoptosis by forming a complex known as the apoptosome. In addition, procaspase-9 contributes to apoptosis. **B** Necroptosis is triggered by LPS through death receptors such as TNFR1/Fas and TLR3/4 in macrophages. **C** Ferroptosis is characterized by iron-dependent massive lipid peroxidation, and Cystine/GSH/GPX4 is a classical ferroptosis inhibition system. **D** In the canonical pyroptosis pathway, DAMP, PAMP, or DNA damage activates caspase-1, which then activates caspase-1 to cleave GSDMD to form GSDMD-N, which binds to membranes and creates pores to induce pyroptosis. In the noncanonical pyroptosis pathway, caspase-4/-5/-11 are activated by direct binding with LPS, which then cleaves GSDMD to induce pyroptosis. *TNFR* Tumor necrosis factor receptors, *TLR* Toll-like receptor, *TDAIL-R* TNF-related apoptosis-inducing ligand receptors, *FADD* Fas-associated death domain protein, *TRADD* TNFR1-associated death domain protein, *TNFα* Tumor necrosis factor-alpha, *TRAIL* TNF-related apoptosis-inducing ligand, *Bax/Bcl-2 RIPK1* Receptor interacting protein kinase 1, *GPX4* glutathione peroxidase 4, *MLKL* Mixed lineage kinase-like, *ROS* Reactive oxygen species; *DAMP* Damage-associated molecular pattern, *PAMP* Pathogen-associated molecular pattern, *AIM2* Absent in melanoma 2, *LPS* Lipopolysaccharide, *GSDMD* Gasdermin D

Similar to pyroptosis, necroptosis is defined by disrupted plasma membrane integrity and the release of cellular contents. Nevertheless, pyroptosis and necroptosis exhibit distinct morphological features. In necroptosis, membrane rupture is explosive, whereas pyroptosis induces cell flattening through plasma membrane leakage [25]. Necroptosis is initiated by death receptors such as TNFR1 and FAS [32, 33]. Ferroptosis represents another form of programmed cell death characterized by iron-dependent massive lipid peroxidation, resulting in membrane damage [34, 35]. Morphologically, ferroptosis is characterized by shrunken mitochondria, increased membrane density, and decreased mitochondrial cristae, culminating in membrane rupture [35–37]. In 2022, Tsvetkov et al. unveiled a novel form of copper-dependent cell death, termed cuproptosis [38]. In this unique mechanism, copper directly binds to lipoylated components of tricarboxylic acid (TCA), inducing aggregation of lipoylated proteins and resulting in the loss of iron-sulfur cluster proteins, ultimately leading to cell death. Importantly, inhibitors targeting apoptosis, necroptosis, ferroptosis, and pyroptosis have proven ineffective in preventing cuproptosis, underscoring its distinct nature as a cell death mechanism [39]. Taken together, we summarized the characteristics of these different forms of cell death in Fig. 2 and Table 1.

**Table 1:** The characteristics of apoptosis, necroptosis, ferroptosis, and pyroptosis

Type of cell death	Morphological characteristics	Inducers	Initiator caspase	Major executor
Apoptosis	Bubbling plasma membrane, shrunkern cells, chromatin aggreegation and condensation	Fas, TNFR, TRAIL-R. TLRs, DNA damage, hypoxia	Caspase-8/10/3/9/6/7	Caspases
Necroptosis	Disrupted plasma membrane integrity, cell contents leakage	TNFR1, Fas, LPS, TLR3/4	Caspase-8/10	RIPK1, RIPK3, MLKL
Ferroptosis	Shrunken mitochondria, increased membrane density and decreased mitochondrial cristae	Fe3 + , cystine, lipid peroxidation,	No	GPX4
Pyroptosis	Cell swelling, pore formation on cell embranes, bubbling and disrupted plasma membrane	DAMPs, PAMPs, acterial infections, LPS, chemotherapy drugs, TNF, GZMA, GZMB	Caspase-1/4/5/11/8	Gasdermins
Cuproptosis	Shrunken mitochondria, disrupted plasma membrane, damage to the endoplasmic reticulum	Cu2 +	No	FDX1

## Molecular mechanisms of pyroptosis

**Fig. 3:**
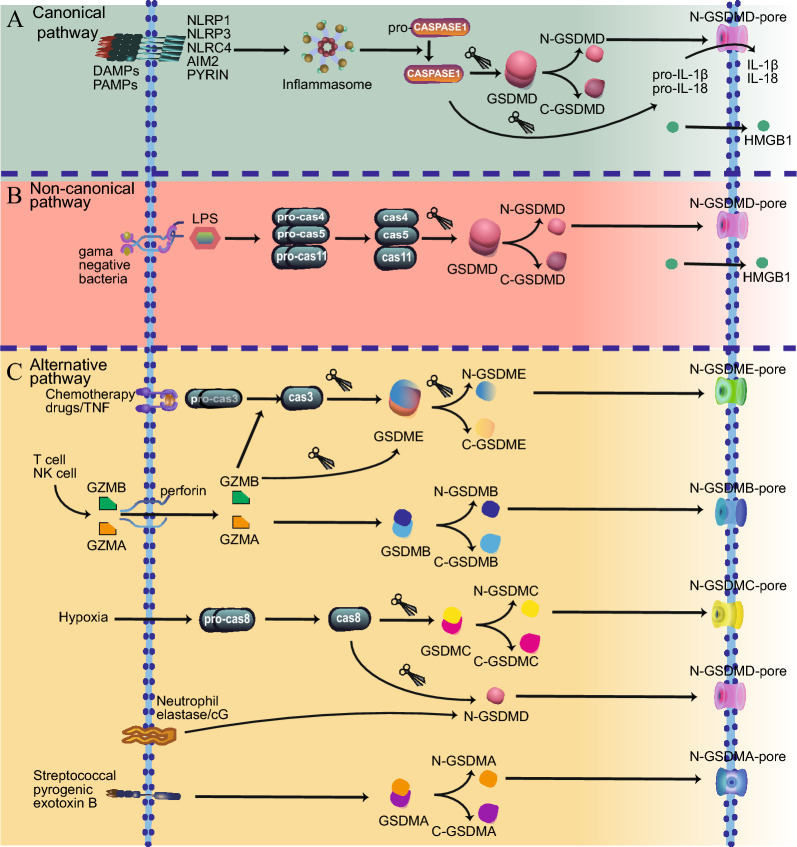
Schematic representation of pyroptosis signaling pathways. **A** In the canonical pathway, PRRs detect DAMPs or PAMPs that stimulate inflammasomes, which then activate caspase-1 to cleave GSDMD to form N-GSDMD and C-GSDMD. Meanwhile, caspase-1 activates pro-IL-1β and pro-IL-18 to form mature IL-1β and IL-18, respectively. The the N-GSDMD domains then bind to the plasma membrane to form pores, which allows IL-1β and IL-18 secretion, eventually resulting in cell swelling and membrane rupture. **B** In the noncanonical pathway, caspase-4/5/11 can directly bind to cytosolic LPS and be activated. Then caspase-4/5/11 cleave GSDMD to promote pyroptosis. **C** In other alternative pathways, chemotherapy drugs or TNF switch apoptosis to pyroptosis through the caspase-3-GSDME axis. GZMB from NK and CD8^+^ T cells can also cleave GSDME directly or by activating caspase-3 to form N-GSDME to induce pyroptosis. GZMA secreted from NK and CD8^+^ T cells promotes GSDMB-mediated pyroptosis. Under hypoxic conditions, GSDMC are cleaved by activated caspase-8. The caspase-8 also cleaves GSDMD in the intestinal epithelial cells to induce pyroptosis. In neutrophils, neutrophil elastase and cathepsin G cleave GSDMD to induce pyroptosis. Streptococcal pyrogenic exotoxin B induces pyroptosis via GSDMA cleavage. *PRRs* Pattern recognition receptors, *DAMPs* Damage-associated molecular patterns, *PAMPs* Pathogen-associated molecular patterns, *GSDMD* Gasdermin D, *GZMB* Granzyme B, *GZMA* Granzyme A, *NK* Natural killer

Pyroptosis is characterized by the formation of cell membrane pores and is a process mediated by the GSDM family [11, 40]. The initiation of pyroptosis involves various pathways, including canonical, noncanonical, and alternative pathways. The GSDM family consists of an N-terminal pore-forming domain and C-terminal autoinhibitory domain connected by a peptide linker [41]. Regardless of the specific pathway, the common molecular mechanism involves the cleavage of GSDMs into N-terminal and C-terminal fragments. Subsequently, the N-terminal domain is incorporated into the cell membrane, forming pores and inducing pyroptosis [40, 42]. In Fig. 3, we summarize the pathways and molecular mechanisms involved in pyroptosis.

### The canonical pathway

In the carcinoma pathway, the activation of caspase-1 is facilitated by inflammasomes, which are multiprotein complexes formed by sensor proteins, adaptor proteins, and effector caspase. The activation of inflammasomes is triggered by pattern recognition receptors (PPRs) detecting both exogenous pathogens and endogenous damage, encompassing bacterial infections, pathogen-associated molecular patterns (PAMPs), and damage-associated molecular patterns (DAMPs) [43, 44]. Numerous PRRs are involved in this process, including NOD-like receptors (NLRs), TLRs, C-type lectin receptors (CLRs), and retinoic acid-inducible gene 1 (RIG-1)-like receptors (RLRs). However, only specific receptors have been identified as direct co-inflammasomes that activate caspase-1, including NLR family pyrin domain-containing (NLRP)1, NLRP3, NLRP4, NLRC4, absent in melanoma 2 (AIM2), TLR4 and Pyrin [45–48]. These receptors interact with the adaptor protein apoptosis-associated speck-like protein containing a caspase recruitment domain (ASC) and induce pro-caspase-1 recruitment and caspase-1 activation via self-cleavage [11].

Some PRRs possess a caspase activation and recruitment domain (CARD), enabling direct recruitment of pro-caspase-1 [49]. Subsequent to activation, caspase-1 cleaves GSDMD at the Asp275 site, generating a 31 kDa N-terminus (N-GSDMD) and a 22 kDa C-terminus (C-GSDMD).Simultaneously, activated caspase-1 processes cytokines such as pro-IL-1β and pro-IL-18 into their mature forms, IL-1β and IL-18 [50]. The N-GSDMD domains then bind to the plasma membrane, forming a pore that allows the release of mature IL-1β and IL-18 to initiate inflammation [51]. Additionally, during cell lysis, IL-1α, inflammasome complexes, and intracellular DAMP such as HMGB1 are also released [11, 52]. Pyroptosis, a form of programmed cell death, plays a crucial role in the context of infections and wounds by activating inflammasomes [53]. In the event of an infection or wound, various pathogens or cellular damage-associated molecules are released, including bacterial components such as LPS and endogenous DAMPs such as ATP or uric acid crystals [54]. Subsequently, NLRs are activated and interact with other proteins to form an inflammasome complex, typically the adaptor protein ASC. The outcome is the initiation of pyroptosis, which leads to the rapid release of pro-inflammatory cytokines and other inflammatory mediators into the extracellular environment. This release attracts immune cells to the site of infection or wound, promoting inflammation, an essential component of the immune response that aids in pathogen elimination and tissue repair in the case of wounds [55]. Moreover, the products of pyroptosis, such as the released DAMPs and pro-inflammatory cytokines, can further stimulate the activation of NLR inflammasomes, creating a positive feedback loop that amplifies the inflammatory response and induces additional rounds of pyroptosis [56]. The interplay between NLR inflammasomes, GSDMs, and pyroptosis is a tightly regulated process aimed at detecting and responding to infections, while preventing excessive tissue damage. Dysregulation of this system can lead to various inflammatory diseases and pathological conditions [57].

### The non-canonical pathway

The non-canonical pathway of pyroptosis operates independently of caspase-1 but relies on caspase-4 and -5 in humans and caspase-11 in mice [58, 59]. These caspases directly bind to LPS from gram-negative bacteria through their CARD domains [44, 59, 60]. Upon binding to LPS, caspase-11 self-cleaves at the D285 site, generating activated caspase-11 species through dimerization [61]. Similarly, caspase-4/5oligomerizes and is activated by LPS [62]. Activated caspases-4/5/11 then cleaves GSDMD into N-GSDMD, forming pores in the cell membrane [9, 10, 40]. Importantly, caspase-4/5/11 could not directly cleave pro-IL-1β and pro-IL-18. Instead, they activate the NLRP3 inflammasome and caspase-1, ultimately leading to the maturation and release of IL-1β/IL-18 through potassium efflux via the GSDMD pores [9, 63]. The endosomal sorting complexes required for transport (ESCRT) machinery, mediated by calcium influx, can repair cell membrane damage caused by GSDMD pores [64]. Consequently, the fate of the cell is influenced to some extent by the number of GSDMD pores and the effectiveness of the membrane repair mechanism. Additionally, activated caspase-11 can cleave pannexin-1, promote the release of cellular adenosine triphosphate (ATP) release and induce pyroptosis by activating the purinergic P2X7 receptor (P2X7R) [65]. This represents a specialized pathway within the noncanonical pathway.

### Alternative pathways

Caspase-3 was initially recognized as the key executor of apoptosis [66]. However, recent research has established that caspase-3 can also play a role in converting apoptosis into pyroptosis by cleaving and activating GSDME under the influence of chemotherapy drugs [67]. This activation results in the accumulation of the N-terminal fragment of GSDME on cellular membranes, forming pores that lead to cell swelling and lysis. Notably, besides chemotherapy drugs, TNF can also shift apoptosis to pyroptosis through the caspase-3-GSDME axis [67]. Although GSDME is not directly involved in the canonical or noncanonical pyroptotic pathway, the N-GSDME fragment cleaved by caspase-3 can activate the canonical pyroptosis pathway, promoting the release of IL-1β/IL-18 [31]. Moreover, in breast cancer cells, PD-L1 has been identified as a factor that switches TNFα-mediated apoptosis to pyroptosis [68]. In a hypoxic environment, p-Stat3 promotes the nuclear translocation of PD-L1 and increases GSDMC expression. Upon tumor necrosis factor-alpha (TNF-α) treatment, caspase-8 cleaves GSDMC and forms an N-GSDMC domain to induce pyroptosis. Additionally, GZMA secreted from cytotoxic T cells and NK cells cleaves GSDMB at the Lys229/Lys244 site, inducing pyroptosis in a caspase-independent manner [20]. Similarly, GZMB from NK cells and CD8^+^ T lymphocytes cleaves GSDME at the caspase-3 site, liberates N-terminal domains (N-GSDME), and forms cell membrane pores [16]. Granzyme-induced pyroptosis transforms non-inflammatory cell death into inflammatory cell death, enhancing inflammatory properties in the tumor microenvironment (TME). Other pathways have been reported to induce pyroptosis. For instance, streptococcal pyrogenic exotoxin B induces pyroptosis via GSDMA cleavage [69], activated caspase-8 cleaves GSDMC in the context of hypoxia [70], and caspase-8 cleaves GSDMD in intestinal epithelial cells to regulate gut homeostasis [71]. Notably, neutrophil-specific serine proteases such as neutrophil elastase and cathepsin G, can cleave GSDMD, generating the N-terminal domain GSDMD-p30, to induce pyroptosis in neutrophils [72, 73].

## Gasdermin family members in cancer progression

**Fig. 4:**
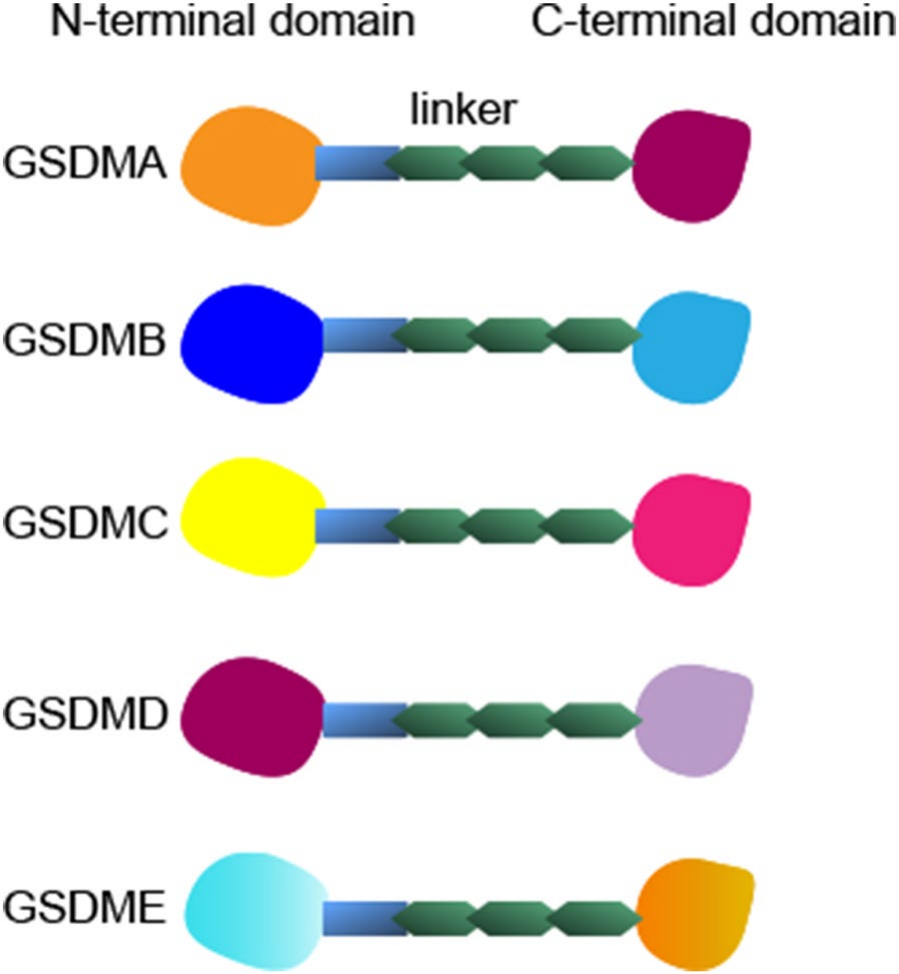
Structural features that gasdermin family members

The GSDM family comprises a group of proteins with shared structural features that play crucial roles in cellular processes such as pyroptosis and inflammation (Fig. 4) [74]. Members of the GSDM family have conserved amino-terminal (NT) and carboxy-terminal (CT) domains connected by a linker region [75]. Notably, PJVK, a member of the GSDM family, has NT and CT domain directly connected to [76]. The NT domain is responsible for executing the cellular functions of GSDMs. Upon activation through proteolytic cleavage, this domain is released and can form pores in the cell membrane. In contrast, the CT domain maintains the repressed state of full-length GDMDs and inhibits the pore-forming ability of NT. This is achieved by masking the NT hydrophobic pocket, which binds lipids [75].

GSDM proteins initially exist in full-length and inactive forms. However, they undergo proteolytic cleavage upon activation by various cellular signals. In activated effector innate immune cells, such as macrophages and dendritic cells, GSDM proteins emerge as central players in the inflammatory response against pathogens [77, 78]. When these immune cells encounter infection or cellular stress, they initiate the production of pro-inflammatory cytokines such as IL-1β and IL-18. This process typically involves the activation of caspase-1 or other caspases that cleave GSDM proteins to release an NT fragment. The NT domain then binds to and punctures the cell membrane, resulting in the formation of pores. These pores disrupt the integrity of the cell membrane, causing cells to swell and eventually burst. The release of cellular contents activates an inflammatory response that recruits immune cells to the site. Furthermore, the pores formed by GSDM proteins facilitate the release of pro-inflammatory cytokines, thereby amplifying inflammatory signaling and enhancing defense against microbial infections. In summary, the primary function of the GSDM family is to mediate pyroptosis, a mechanism that eliminates infected or damaged cells and initiates an inflammatory response to protect surrounding tissue. This process is crucial for the body's defense against pathogens and maintenance of tissue homeostasis. However, it’s important to note that excessive pyroptosis can lead to tissue damage and contribute to the pathogenesis of certain diseases [79].

The regulation of GSDMs expression can vary across different conditions, and the specific circumstances that leading to the upregulation of GSDM expression can be complex. For instance, studies have reported the upregulation of GSDMB in breast cancer, and its expression has been associated with the progression and poor prognosis of breast cancer [80]. GSDME expression was found to be upregulated in colorectal cancer. Increased expression of GSDME has been linked to enhanced pyroptotic activity, and it may contribute to the inflammatory response associated with colorectal cancer progression [81]. Indeed, some studies have proposed that GSDME is often silenced by methylation in breast cancer. Nevertheless, researchers have explored the re-expression of GSDME as a potential strategy to induce pyroptosis in breast cancer cells [82]. Modulation of GSDM expression can also be influenced by interactions with immune cells within the TME. The infiltration of cytotoxic T cells and NK cells, which recognize and eliminate cancer cells expressing GSDMs, may affect the overall expression pattern [83].

The GSDM family plays a crucial role in maintaining tissue homeostasis and has been implicated in various physiological and pathological processes, including tumorigenesis and cancer progression. Here, we summarize the role of GSDMs in cancer progression.

### The role of GSDMA in cancer progression

GSDMA is predominantly expressed in epithelial tissues. In the skin, its expression is notable in keratinocytes, the predominant cell type in the epidermis [84, 85]. GSDMA has also been detected in the gastrointestinal tract, specifically in the stomach and intestines. However, it is silenced in most GC cell lines [69, 86] (Table 2). Previous studies have indicated that GSDMA plays a role in gastric cell apoptosis and is a potential target of LIM domain only 1 (LMO1) in transforming growth factor-beta (TGF-β)-induced apoptosis [86]. Therefore, restoring GSDMA expression in gastric cancer cells may be an effective strategy for gastric cancer treatment. Conversely, GSDMA is overexpressed in ovarian cancer tissues compared to normal tissues and is associated with a poorer prognosis [12]. Hence, GSDMA serves as a protumorigenic factor in ovarian cancer. However, the precise mechanisms underlying GSDMA-induced cell death remain unclear. Shi et al. discovered that GSDMA3 could not be cleaved by inflammatory caspases. However, artificial interdomain cleavage of GSDMA3 could induce cell pyroptosis [10]. Interestingly, overexpression of the GSDMA3-N terminal domain enhanced cell pyroptosis, a phenomenon not observed in full-length GSDMA3. Moreover, the C-terminal domain of GSDMA3 can co-precipitate with the N-terminal domain and reverse GSDMA3-N-induced cell autophagy [87]. Consequently, GSDMA3 exhibits an autoinhibited structure akin to other gasdermins [10]. GSDMA3 mutants lose their autoinhibition ability and become activated, leading to pyroptosis [10].

**Table 2:** Features and functions of gasdermin family

Human gasdermin	Mouse gasdermin	Activated by	Tissue expression	Related cancers
GSDMA	Gsdma1, Gsdma2, Gsdma3	streptococcal pyrogenic exotoxin B	Skin, gastrointestinal tract	Gastric cancer, ovarian cancer
GSDMB	N	GZMA, GZMB, caspase3/6/7	Normal tissues and tumor cells	Gastric cancer, cervical cancer, hepatocarcinoma, bladder cancer, clear cell renal cell carcinoma, bladder cancer
GSDMC	Gsdmc1, Gsdmc2, Gsdmc3, Gsdmc4	Caspase8	Melanoma cells	Melanoma, lung adenocarcinoma, breast cancer, gastric cancer, colorectal cancer, kidney clear cell cancer
GSDMD	Gsdmd	Caspase1/4/5/8/11, neutrophil elastase, cathepsin G	Nucleus, plasma membrane	Hepatocellular carcinoma, non-small lung cancer, gastric cancer,
GSDME	Gsdme	Caspase3	Plasma membrane, mitochondrial	Breast cancer, lung adenocarcinoma, oesophageal squamous cell carcinoma, oral squamous cell carcinoma, colorectal cancer,

### The role of GSDMB in cancer progression

GSDMB is widely expressed in both normal tissues and various tumor cells, including gastric cancer [88], cervical cancer [89], breast cancer [90], and hepatocarcinoma [91] (Table 2). In the gastrointestinal tract, GSDMB is found in the stomach, small intestine, and colon. Despite extensive investigation in numerous studies, the precise role of GSDMB in tumorigenesis and its associated mechanisms remain elusive. Notably, GSDMB is markedly overexpressed in bladder cancer and clear cell renal cell carcinoma, correlating with poor prognosis [13, 92]. Recent studies has identified ubiquitin-specific peptidase 24 (UP24), a deubiquitinating enzyme, as a regulator of GSDMB stability. UP24 deubiquitinates GSDMB, thereby reducing its degradation. Consequently, stabilized GSDMB has been shown to modulate glucose metabolism by enhancing signal transducer and activator of transcription 3 (STAT3) phosphorylation in bladder cancer cells [13]. This highlights the potential of GSDMB as a promising target for tumor treatment.

Research indicates that GSDMD is cleaved by inflammatory caspases, whereas GSDMB is cleaved by apoptotic executioner caspases, includingcaspase-3, caspase-6, and caspase-7, [10, 93]. In addition to caspases, GSDMB is susceptible to cleavage by GZMA, which originates from NK cells and cytotoxic T lymphocytes (CTLs) [10, 20]. The induction of pyroptosis by GSDMB through NK cells operates independently of caspases and can be effectively inhibited by blocking the perforin-granzyme signaling pathway in human 293 T cells. Notably, cleavage of GSDMB by observed to converts GZMA-induced apoptosis to pyroptosis. In a mouse model, Zhou et al. observed no discernible difference in tumor growth between GSDMB^+^ and GSDMB^−^ tumor grafts. However, when GSDMB expression was combined with anti-PD-1 therapy, significant inhibition of tumor growth was observed in CT26 cells [20]. This suggests that the synergy between GSDMB expression and anti-PD-1 therapy holds promise for impeding tumor progression. Consequently, GZMA-GSDMB-induced pyroptosis has emerged as a potential focal point for enhancing antitumor immunotherapy. In addition to its role in mediating pyroptosis, GSDMB can also induce non-canonical pyroptosis by cleaving GSDMD and binding with caspase-4 through the N-terminal region spanning amino acids 1–83 [94]. Unlike the N-terminals of GSDMD and GSDMA, which form pores in the cell membrane leading to pyroptosis and cell death, the GSDMB-N terminal associates with sulfatide instead of cardiolipin, rendering it incapable of inducing cell death [93].

### The role of GSDMC in cancer progression

Recent research on the biological function and disease pathogenesis of GSDMC is limited. Initially identified in metastatic melanoma cells, GSDMC was named melanoma-derived leucine zipper-containing extranuclear factor (MLZE) [11, 95]. Elevated GSDMC expression has been associated with poor prognosis in various cancers, including lung adenocarcinoma [96], breast cancer [68], gastric cancer [97], colorectal cancer [98], and clear cell renal cell carcinoma [14] (Table 2). This suggests a potential oncogenic role for GSDMC in cancer progression. Additionally, Hou et al. found that TNF-α-induced apoptosis can transition to pyroptosis by activating GSDMC expression [68]. Under hypoxic conditions, PD-L1 undergoes nuclear translocation and forms a complex with p-Y705-Stat3. This complex promotes GSDMC expression, which is subsequently cleaved by caspase-8. Subsequently, the GSDMC-N terminal domain forms a pore by binding to the cell membrane and inducing pyroptosis. Notably, pyroptosis induced by GSDMC in hypoxic regions promotes tumor development and suppresses the antitumor immune response [68].

In melanoma cells, the metabolite α-ketoglutarate (α-KG), known to induce pyroptosis through GSDMC, inhibits tumor progression and metastasis [70]. α-KG, a crucial intermediate metabolite of the tricarboxylic acid (TCA) cycle, plays a pivotal role in metabolic homeostasis, protein modification, tumorigenesis, and cell death [99–101]. It achieves his by elevating reactive oxygen species (ROS) levels, leading to the oxidation of the plasma membrane-localized death receptor 6 (DR6). This, in turn, recruit caspase-8 to cleave GSDMC to cleave caspase-8 to cleave GSDMC, ultimately inducing pyroptosis. Importantly, this process was further enhanced in an acidic environment [70]. In summary, targeted modulation of α-KG-induced pyroptosis is a promising strategy for tumor treatment.

### The role of GSDMD in cancer progression

GSDMD is widely expressed in various tissues, including immune cells such as macrophages, where it plays a pivotal role in pyroptosis [102]. Despite extensive research, the specific roles and mechanisms of GSDMD in cancer remain elusive. Research has revealed that GSDMD play a dual role in cancer. On one hand, its overexpression promotes tumor cell death; on the other hand, it is associated with poorer prognosis in certain malignant tumors (Table 2). For instance, GSDMD is upregulated in hepatocellular carcinoma [103] and non-small cell lung cancer [104], with higher levels of GSDMD expression in tumor tissues predicting a less favorable patient survival outcome. Moreover, GSDMD expression levels were correlated with tumor size and stage in NSCLC. Knockdown of GSDMD has been demonstrated to suppress tumor progression by promoting the mitochondrial apoptotic pathway and inhibiting the epidermal growth factor receptor (EGFR)/Akt pathway [104]. In contrast, GSDMD was found to be downregulated in gastric cancer compared to that in adjacent normal tissue. Reduced expression of GSDMD was identified as a factor contributing to the progression of gastric cancer by facilitating the transition from the S phase to the G2/M phase [105].

Moreover, GSDMD localization of serves as a prognostic marker for malignant tumors. Notably, GSDMD is expressed not only in the cytoplasm, but also in the nucleus. Interestingly, nuclear GSDMD, as opposed to its cytoplasmic counterpart, has been found to suppress the growth of colorectal cancer cells by inducing apoptosis under chemotherapy stimulation rather than through pyroptosis-mediated cell death. The loss of GSDMD expression in the nucleus localization has been correlated with a worsened prognosis [106]. Furthermore, the subcellular localization of GSDMD expression also affects immune cell infiltration. Membranous GSDMD expression is associated with CD68^+^ macrophages in the tumor center and CD8^+^ T cells in the tumor front, whereas nuclear GSDMD exhibits the opposite association [107].

In addition to its impact on cancer cells, GSDMD also affects immune cells. Analysis of The Cancer Genome Atlas (TCGA) database revealed a positive correlation between GSDMD expression and the levels of CD8^+^ T cell markers in various types of tumors [108]. The mRNA levels of GSDMD were elevated in activated CD8^+^ T cells, accompanied by an increase in GSDMD cleavage. Notably, GSDMD deficiency impairs the cytotoxic function of CD8^+^ T cells. This suggests a supportive role for GSDMD in the tumor-killing effect of CD8^+^ T cells. However, Jiang et al. made contrasting observations, noting the high expression of GSDMD in antigen-presenting cells (APCs) within the TME. In this context, GSDMD expression in APCs was found to inhibit the antitumor effect of anti-PD-L1 therapy. Conversely, GSDMD deficiency in APCs enhances the anti-tumor response and promotes the activation of CD8^+^ T cells [109].

### The role of GSDME in cancer progression

GSDME is expressed in various tissues, including the skin and gastrointestinal tract [110]. It is also present in the inner ear and its activation has been linked to hearing loss. The status of GSDME expression in tumors is currently a subject of controversial. While certain studies suggest that GSDME is downregulated in tumors compared to normal tissue [111–113], others have reported the opposite observation [114]. Additionally, some studies have found no discernible differences in GSDME expression between cancer and normal tissues [115, 116]. Hence, GSDME expression cannot reliably serve as a predictor for cancer detection. Notably, there is a negative correlation between GSDME expression and estrogen receptor levels in breast cancer, leading to its designation as inversely correlated with estrogen receptor expression (ICERE-1) [111, 117]. Additionally, GSDME expression was higher in lobular adenocarcinomas than in ductal adenocarcinomas. In lung adenocarcinomas, GSDME expression was correlated with EGFR, STK11, and KEAP1/NFE2L2 mutations. Specifically, it is upregulated in EGFR-mutant tumors but downregulated in neoplasm STK11- or KEAP1/NFE2L2-mutations [116]. In esophageal squamous cell carcinoma, GSDME expression is notably elevated in cancer tissues compared to that in normal tissues. Notably, patients with high GSDME expression levels demonstrated a better five-year survival rate than those with low GSDME expression levels [114]. As such, GSDME emerges as a valuable prognostic marker in esophageal squamous cell carcinoma. Furthermore, Wang et al. made similar observations in oral squamous cell carcinoma, where GSDME expression levels in tumor tissues surpassed those in metastatic lymph nodes. High GSDME expression is associated with a more favorable prognosis and enhanced anti-tumoral immunity [118]. Additionally, GSDME-mediated pyroptosis potentiates the antitumor effect induced by cisplatin.

Unlike gene expression patterns, researchers have identified GSDME methylation as a potential marker for cancer detection and prognosis prediction across various tumor types [119]. For instance, in breast cancer, tumor tissue exhibits higher methylation levels in clinical practice guidelines (CpGs) located in the gene promoter than normal breast tissue. Conversely, the gene body displays opposite methylation pattern [111]. Moreover, lobular adenocarcinomas demonstrated elevated GSDME promoter methylation values compared with ductal adenocarcinomas. Remarkably, GSDME gene body methylation, as opposed to the promoter, exhibited an inverse correlation with the 5-year overall survival time, specifically in ductal adenocarcinomas [111]. Hence, GSDME methylation has emerged as a valuable prognostic indicator for breast cancer. However, no significant correlation was observed between GSDME methylation and 5-year survival [115]. Nevertheless, GSDME promoter methylation was higher in colorectal cancer cases characterized by lymphatic vessel invasion and high tumor-node-metastasis (TNM) stage. Furthermore, increased methylation was identified in right-sided colorectal cancer compared to that in left-side [115, 120]. Consequently, GSDME methylation holds promise as a marker for colorectal cancer detection.

Moreover, GSDME plays a crucial role in the TME regulation. Specifically, wild-type GSDME expression enhances phagocytosis by tumor-associated macrophages (TAMs) and promotes increased infiltration of immune cells, including CD8^+^ T cells and NK cells. mutant GSDME loses this regulatory function [16]. Additionally, the antitumor effect of GSDME was notably absent in mice lacking mature lymphocytes. This underscores that GSDME’s tumor-suppressive function of GSDME is mediated through pyroptosis-induced activation of antitumor immunity [16]. Single-cell RNA sequencing has revealed that treatment-induced pyroptosis in 4T1 cells is accompanied by augmented infiltration of CD4^+^ and CD8^+^ T cells, NK cells, and polarization towards M1 macrophages. Conversely, there was a decrease in the number of monocytes, neutrophils, and myeloid-derived suppressor cells [121]. Therefore, the induction of pyroptosis holds promise as a strategic approach to turn convert “cold” tumors into “hot” tumors.

## The immune-modulatory effects of pyroptosis

**Fig. 5:**
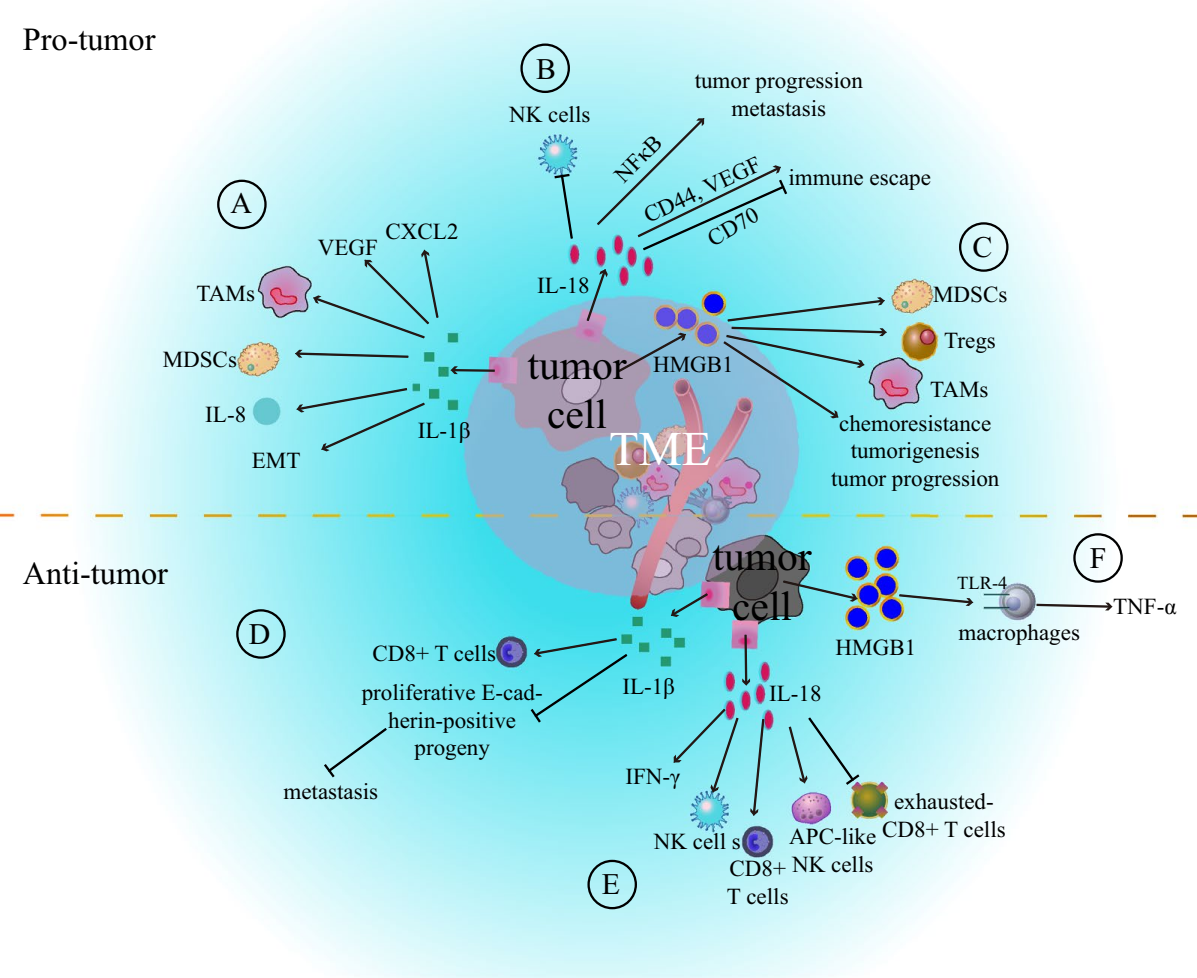
Regulatory role of pyroptosis in the tumor microenvironment. In several cancers, pyroptosis promotes tumor progression by inducing a pro-tumor immune microenvironment via inflammatory cytokines, such as (**A**) IL-1β, (**B**) IL-18, and (**C**) HMGB1. However, pyroptosis induces an anti-tumor immune microenvironment in some other cancers in the same manner (**D**–**F**). *MDSC* Myeloid-derived suppressor cell, *TAMs* Tumor-associated macrophages, *NK* Natural killer, *Tregs* Regulatory T lymphocytes

As a form of inflammatory cell death (ICD), pyroptosis has the potential to transform the immune “cold” tumors into “hot” tumors by releasing proinflammatory factors and reshaping immune cells within the TME [16]. One of the distinctive features of pyroptosis is the release of inflammatory cytokines, including IL-1β, IL-18, and HMGB1 [15, 23, 122]. IL-1β and IL-18 are secreted through the GSDMD-forming pores, whereas HMGB1 is released after pyroptosis-induced cell lysis [7, 11, 52]. These inflammatory cytokines, particularly IL-1β and IL-18, play crucial roles in both innate and adaptive immunity [123]. Thus, pyroptosis emerges as a vital process that bridges the connection between innate and adaptive immunity. A deeper understanding of the mechanisms underlying pyroptosis and its impact on TME reprogramming may pave the way for innovative targeting strategies for future therapeutic approaches (Fig. 5).

### Pyroptosis-related cytokine IL-1β

The role of IL-1β in tumor progression remains controversy [124]. Although IL-1β is well recognized for its tumor-promoting function [125], its specific impact can vary across different cancers. For instance, IL-1β has been shown to promote gastric cancer initiation and progression by inducing the recruitment and activation of myeloid-derived suppressor cells (MDSC) through the IL-1R/NF-κB pathway [126]. Studies using IL-1R-deficient mice have demonstrated a hindrance in MDSC accumulation, thereby inhibiting tumor progression in breast cancer [127]. In the 4T1 tumor model, IL-1β was found to promote the differentiation of tumor-infiltrating inflammatory monocytes into macrophages by inducing the production of chemokine (CC-motif) ligand 2 (CCL2) [128]. Interestingly, the deletion of IL-1β not only inhibited macrophage recruitment but also enhanced CD8^+^ T cell activation, thereby promoting a robust antitumor response. Simultaneously, a combination therapy involving anti-PD-1 and anti-IL-1β demonstrated enhanced therapeutic efficacy. IL-8 has been implicated in promoting tumor progression and metastasis in various cancers, including prostate, breast, and gastric cancer [129–131]. However, the precise underlying mechanisms require further investigation. Notably, Olivera et al. revealed that IL-1β and TNFα stimulate the production of IL-8, subsequently attracting immunosuppressive myeloid cells to the TME. Effective blocking of IL-1β and TNF-α has proved to be a successful strategy for halting the pro-tumorigenic microenvironment [132]. Furthermore, IL-1β was found to be highly expressed in Kirsten rat sarcoma viral oncogene homolog (KRAS) -mutant lung cancer, IL-1β blockade demonstrated a transformative effect, switching the immune-suppressive TME to an anti-tumor immune state. This was achieved through the increased infiltration of CD8^+^ T cells and simultaneous suppression of neutrophils and MDSCs [133].

Furthermore, IL-1β plays a crucial role in the promotion of tumor metastasis., Primary contribution to treatment failure [134]. Epithelial-mesenchymal transition (EMT) has been identified as a key step in this process [135]. Previous studies have established that both IL-1β and IL-18 play essential roles in fostering cancer cell metastasis [136, 137]. For example, Li and colleagues discovered that IL-1βenhances the invasiveness and proliferation of colon cancer cells by promoting the EMT phenotype, achieved through the upregulation of activator Zeb1 expression [138]. Furthermore, IL-1β was identified as a promoter of vascular endothelial growth factor (VEGF) and C-X-C motif chemokine ligand 2 (CXCL2) expression, which are both crucial for tumor growth and metastasis [139]. In contrast, a recent study found that IL-1β may hinder breast cancer metastasis by inhibiting the production of proliferative E-cadherin-positive progeny derived from metastasis-initiating cancer cells [140]. Intriguingly, a higher expression level of IL-1β was associated with better prognosis and distant metastasis-free survival. Therefore, IL-1β may exhibit distinct roles in different tumors, and further investigation is required to elucidate the underlying molecular mechanisms.

### Pyroptosis-related cytokine IL-18

As a member of the IL-1 family of cytokines, IL-18 has been established as a pivotal regulator of the activation and differentiation of immune cells [141]. In collaboration with IL-12 and IL-15, IL-18 promotes the activation of memory-like NK cells, subsequently increasing the secretion of interferon-gamma (IFN-γ) [142]. Additionally, IL-18 induces the expression of HLA-DR, HLA-DQ, CD80, and CD86 in NK cells, directing them toward an APC-like phenotype [143]. However, studies have also found that IL-18 does not effectively enhance the antitumor effect in melanoma [144]. Zhou et al. revealed that the high-affinity IL-18 decoy receptor, IL-18 binding protein (IL-18BP), is markedly upregulated and interacts with IL-18, thereby diminishing its antitumor activity [145]. In response, they engineered a ‘decoy-resistant’ IL-18 (DR-18) designed to resist inhibition by IL-18BP. DR-18 demonstrates robust antitumor effects when compared to wild-type IL-18, achieved through the activation of effector CD8^+^ T cells and a reduction in exhausted CD8^+^ T cell numbers. Additionally, DR-18 enhances the efficacy of anti-PD-1 treatment by promoting the activation and maturation of NK cells [145]. Therefore, targeting IL-18BP may be an effective strategy for tumor treatment.

In contrast, IL-18 induces differentiation of naïve T cells into Th2 cells by promoting the production of IL-4 [146]. Terme et al. reported that low-level IL-18 disrupts the NK cell arm of tumor immunosurveillance by increasing PD-1 expression [147]. Additionally, IL-18 transforms Kit (-) CD11b (-) NK cells into Kit ( +) NK cells, leading to upregulation of B7-H1/PD-L1 expression. These cells infiltrate lymphoid organs and exert an immunoablative effect [148]. Thus, IL-18 plays diverse roles in the regulation of tumor progression. Furthermore, IL-18 is overexpressed in certain tumors and is associated with poor prognosis, as observed in pancreatic cancer, renal cell carcinoma, and extranodal natural killer/T-cell lymphoma [149–151]. In pancreatic cancer, IL-18 promotes tumor progression and metastasis through the NF-κB signaling pathway, with increased NF-κB perpetuating elevated IL-18 expression and forming a positive feedback loop [149, 152]. Considering the established roles of CD70, CD44, and VEGF in immune escape, Kang et al. found that IL-18 is involved in this process by suppressing CD70 and increasing CD44 and VEGF in stomach cancer [153–157]. In summary, the characteristics of IL-18 in the TME and tumor progression make it an attractive target for cancer therapy.

### Pyroptosis-related cytokine HMGB1

Unlike IL-18 and IL-1β, HMGB1 is released through pyroptosis-induced cellular lysis rather than gasdermin-mediated pore formation [52]. HMGB1’s role in tumor development is multifaceted and serves as a double-edged sword. HMGB1 functions as a tumor promoter [158–161]. For example, GSDME-mediated pyroptosis contributes to tumorigenesis and promotes tumor progression in colitis-associated colorectal cancer through the release of HMGB1. This, in turn, activates the extracellular signal-related kinase 1 and 2 (ERK1/2) pathway [15]. Furthermore, LPS-induced expression of the pro-inflammatory cytokines IL-6, TNF-α, and IL-1β is mediated by HMGB1. Inhibition of HMGB1 has shown significant efficacy in reducing the incidence of inflammatory responses, making it a potential target for treating colon cancer [162]. HMGB1 also plays a crucial role in chemoresistance. Cancer-associated fibroblasts have been identified as promoters of tumor metastasis in NSCLC and inducers of doxorubicin resistance in breast cancer by increasing HMGB1 production [158, 159]. Anti-HMG1 antibodies have been shown to be effective in restoring sensitivity to chemotherapy. Additionally, blocking HMGB1 has the potential to reshape the TME and enhance the efficacy of immune checkpoint inhibitors [163]. Hubert’s findings indicate that HMGB1 inhibitors reduce the population of MDSC and regulatory T lymphocytes (Tregs), increase the M1/M2 ratio, and augment dendritic cell activation. On the contrary, HMGB1 also exhibits antitumor effects. Kang et al. revealed that HMGB1 suppresses pancreatic ductal adenocarcinoma tumorigenesis driven by oncogenic K-Ras. Deletion of HMGB1 induces the release of inflammatory nucleosomes, disrupting their suppressive effects [164]. Furthermore, HMGB1 secreted by irradiated cancer cells stimulates macrophages to produce TNF-α via the TLR-4 signaling pathway, inhibiting tumor progression and metastasis [165, 166].

### Pyroptosis effects on immune cells

Tumor suppression primarily relies on cytotoxic lymphocyte killing, with pyroptosis emerging as a critical mechanism by which cytotoxic lymphocytes exert anti-tumor effects. Pyroptosis in tumor cells contributes to the reprogramming of the TME into an immunostimulatory state. Experimental evidence supports the hypothesis that caspase-3 cleaves GSDME, and induces pyroptosis. Additionally, GZMB can induce pyroptosis and enhance anti-tumor immunity by directly cleaving GSDME at the caspase-3 site [16, 67]. Furthermore, non-cleavable or pore-defective GSDME loses its tumor-suppressive function, underscoring the importance of pyroptosis in activating antitumor immunity. Moreover, the overexpression of GSDME enhances the phagocytic capacity of TAMs and increases both the number and antitumor activity of tumor-infiltrating CD8^+^ T lymphocytes and NK cells [16]. GSDME transforms non-inflammatory apoptotic cell death into inflammatory pyroptosis cell death, effectively activating anti-tumor immunity. A novel sonodynamic-immunomodulatory strategy utilizing a LY364947-loaded porous coordination network (PCN-224) has demonstrated the ability to remodel the TME and enhance tumor immunotherapy by inducing pyroptosis [167]. Under ultrasound irradiation, the sonosensitizer generates reactive oxygen species and activates caspase-3, subsequently activating GSDME to induce pyroptosis. As previously mentioned, pyroptosis plays a crucial role in mediating antitumor immune responses. However, the extracellular matrix (ECM), a major component of the TME, is characterized by high collagen density and increased stiffness, contributing to an immunosuppressive TME by inhibiting the recruitment, proliferation, and activation of immune cells [168, 169]. LY364947 effectively loosened ECM structure by depleting collagen. This leads to dendritic cell maturation, CD8^+^ T cell infiltration, and memory T cell proliferation. Ultimately, tumors were completely eradicated in the 4T1 mouse model [167].

## Potential strategies targeting pyroptosis

**Fig. 6:**
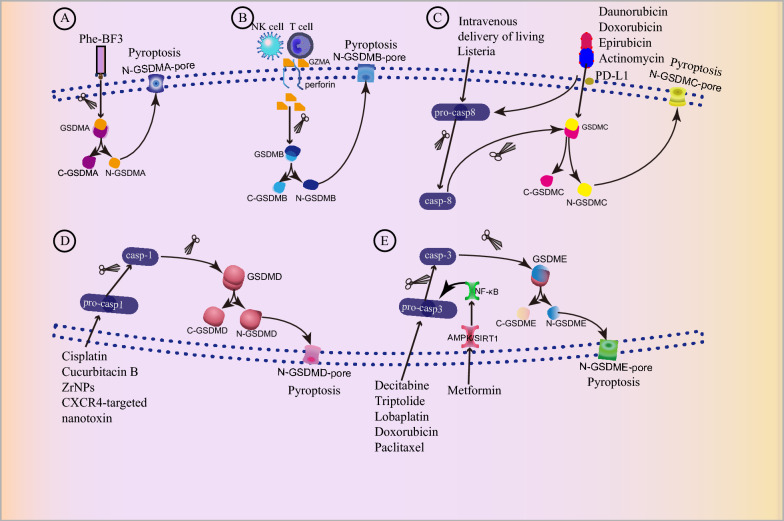
Potential strategies for targeting pyroptosis in cancer treatment. **A** A bioorthogonal chemical system in which cancer-imaging Phe-BF3 selectively cleaves GSDMA3 to induce pyroptosis. **B** NK cells and CTL-derived GZMA cleave GSDMB to induce pyroptosis. **C** Intravenous delivery of live Listeria activates caspase-8 and cleaves GSDMC. Although almost all chemotherapy drugs can promote GSDMC expression and nPD-L1 translocation, only antibiotics, such as daunorubicin, doxorubicin, epirubicin, and actinomycin-D, have been demonstrated to activate caspase-8 and then cleave GSDMC to induce pytoptosis initiation in cancer cells. **D** Cisplatin, Cucurbitacin B, ZrNPs, and CXCR4-targeted nanotoxins induce cancer cell pyroptosis by activating the caspase-1/GSDMD pathway. **E** Chemotherapeutic drugs switch from apoptosis to pyroptosis by activating caspase-3 to cleave GSDME. *Phe-BF3* Phenylalanine trifluoroborate, *GSDMA* Gasdermin A, *NK* Natural killer, *CTLs* cytotoxic T lymphocytes, *GZMA* Granzyme A, *GSDMB* Gasdermin B, *GSDMC* Gasdermin C, *ZrNPs* K3ZrF7: Yb/Er upconversion nanoparticles

As gasdermins and inflammatory cytokines involved in pyroptosis play an important role in the occurrence and development of tumors, researchers have investigated agents targeting these gasdermins (Fig. 6) and cytokines. The agents reported to be useful in preclinical and clinical experiments are listed in Tables 3 and 4.

**Table 3:** Potential strategies targeting gasdermins for cancer treatment

Targets	Agents	Mechanism	Tumor cells type	References
GSDMA3	A bioorthogonal chemical system	The Phe-BF3 selectively cleaved GSDMA3	Hela cell, EMT6, 4T1	[133]
GSDMB	Nanocapsule loaded with an anti-GSDMB antibody	Inhibition the expression of GSDMB	Breast cancer	[183]
GSDMC	Listeria	Increase GSDMC expression, recruit immune cells	Colorectal cancer	[185]
	Daunorubicin, epirubicin, actinomycin-D	Increase GSDMC expression,	Breast cancer	[53]
GSDMD	Cisplatin	Activate caspase-1/GSDMD pathway, increase MEG3 expression	Breast cancer	[187]
	Cucurbitacin B	Bind to TLR4 and increase ROS inductiona and cytosolic calcium release	Non-small cell lung cancer	[191]
	ZrNPs	Induce GSDMD cleavage, activate caspase-1	Breast cancer	[192]
	CXCR4-targeted nanotoxin	Interact with CXCR4 receptor	Colorectal cancer	[193]
	Dimethyl fumarate	Inhibit GSDMD	Hepatocellular carcinoma	[116]
GSDME	Decitabine	Upregulate GSDME expression and switch apoptosis to pyroptosis	Breast cancer	[24]
	Triptolide	Activate caspase-3	Head and neck cancer	[33]
	Lobaplatin	Activate caspase-3	Colon cancer	[197, 198]
	Cisplatin	Activate caspase-3	Non-small cell lung cancer	[199]
	Carrier-free nanoplatform assembled with cytarabine and chlorin e6	Accumulate ROS and induce immunogenic cell death, release HMGB1, ATP and calcitonin, activate GSDME	Breast cancer	[200]
	BNP	Activate caspase-3, promote cytochrome c release, increase GSDME expression	Breast cancer	[203]
	CXCR4-targeted nanotoxins	Activate caspase-3	Head and neck cancer	[204]
	Nanoruner platform	Activate phospholipase C signalling transduction in early endosomes		[209]
	Metformin	Activate AMPK/SIRT1 signaling, promote NF-κB expression	Hepatoblastoma, colon cancer, breast cancer

**Table 4:** Agents targeting on inflammatory cytokines in clinical trials

Targets	Drugs	Phase	Conditions	Sponsor	Gov identifier
IL-1β	Canakinumab	3	Non-Small-Cell Lung	Novartis Pharmaceuticals	NCT03626545
IL-1β	Canakinumab	3	Non-Small-Cell Lung	Novartis Pharmaceuticals	NCT03631199
IL-1β	Canakinumab	2	Chronic Myelomonocytic Leukemia	M.D. Anderson Cancer Center	NCT04239157
IL-1β	Canakinumab		Myelodysplastic Syndrome		
IL-1β	Canakinumab		Recurrent Chronic Myelomonocytic Leukemia		
IL-1β	Canakinumab		Recurrent Myelodysplastic Syndrome		
IL-1β	Canakinumab		Refractory Chronic Myelomonocytic Leukemia		
IL-1β	Canakinumab		Refractory Myelodysplastic Syndrome		
IL-1β	Canakinumab	1	Metastatic Pancreatic Ductal Adenocarcinoma	Pancreatic Cancer Action Network	NCT04581343
IL-1β	Canakinumab	3	Lung Cancer	Mario Negri Institute for Pharmacological Research	NCT05725343
IL-1β	Canakinumab	2	Non-small Cell Lung Cancer	Memorial Sloan Kettering Cancer Center	NCT04905316
IL-1β	Canakinumab	3	Non-small Cell Lung Cancer	Novartis Pharmaceuticals	NCT03447769
IL-1β	Canakinumab	2	Non-small Cell Lung Cancer	Novartis Pharmaceuticals	NCT03968419
IL-1β	Canakinumab	2	Lung Carcinoma	M.D. Anderson Cancer Center	NCT04789681
IL-1β	Canakinumab	1	Triple Negative Breast Cancer	Novartis Pharmaceuticals	NCT03742349
IL-1β	Canakinumab	3	Metastatic Pancreatic Cancer	Pancreatic Cancer Action Network	NCT04229004
IL-1β	Canakinumab		Metastatic Pancreatic Adenocarcinoma		
IL-1β	Canakinumab	1	Non-small Cell Lung Cancer	Novartis Pharmaceuticals	NCT03064854
IL-1β	Canakinumab	2	Melanoma	Novartis Pharmaceuticals	NCT03484923
IL-1β	Canakinumab	3	Non-small Cell Lung Cancer	Novartis Pharmaceuticals	NCT03631199
IL-1β	Genakumab	1	Malignant Solid Tumors	GeneScience Pharmaceuticals Co., Ltd	NCT05441046
IL-1R	Anakinra	2	Multiple Myeloma	Radboud University Medical Center	NCT03233776
IL-1R	Anakinra	2	Multiple Myeloma	Radboud University Medical Center	NCT04099901
IL-1R	Anakinra	2	Pancreatic Adenocarcinoma	Baylor Research Institute	NCT04926467
IL-1R	Anakinra	1	Metastatic Breast Cancer	Baylor Research Institute	NCT01802970
IL-1R	Anakinra	1	Rectal Cancer	Goethe University	NCT04942626
IL-1R	Anakinra	2	Metastatic Colorectal Cancer	Centre Georges Francois Leclerc	NCT02090101
IL-1R	Anakinra	1	Advanced Malignant Neoplasm	M.D. Anderson Cancer Center	NCT01624766
			Metastatic Malignant Neoplasm		
			Recurrent Malignant Neoplasm		
			Refractory Malignant Neoplasm		
IL-1R	Anakinra	1	Pancreas Cancer	Baylor Research Institute	NCT02021422
IL-1R	Anakinra	2	Multiple Myeloma and Plasma Cell Neoplasm	Mayo Clinic	NCT00635154
IL-1R	Anakinra	1	Pancreatic Adenocarcinoma	Baylor Research Institute	NCT02550327
IL-1R	Anakinra	2	Multiple Myeloma	Radboud University Medical Center	NCT03233776
IL-1R	Anakinra	2	Pancreatic Adenocarcinoma	Baylor Research Institute	NCT04926467
IL-1R	Anakinra	2	Diffuse Large B-Cell Lymphoma	Jonsson Comprehensive Cancer Center	NCT04205838
IL-1R	Anakinra		High Grade B-Cell Lymphoma		
IL-1R	Anakinra		Progressive Disease		
IL-1R	Anakinra		Recurrent Diffuse Large B-Cell Lymphoma		
IL-1R	Anakinra	2	B Cell ALL	Memorial Sloan Kettering Cancer Center	NCT04148430
IL-1R	Anakinra		B-Cell Lymphoma		
IL-1R	Anakinra		B-cell Non Hodgkin Lymphoma		

### Potential strategies targeting gasdermins

GSDM^NT^, known to trigger pyroptosis and elicit antitumor immune responses, has emerged as a highly promising strategy for anticancer therapy. Given its broad cytotoxicity in mammalian cells, the production and delivery of GSDM^NT^ into cancer cells is challenging. Lu et al. constructed a recombinant adeno-associated virus expressing GSDM^NT^ [170]. They used the mammal-specific promoter to drive GSDM^NT^ expression and packed the virus into insect cells to avoid its expression, meanwhile, recombinant adeno-associated virus-Cre was employed to recover the expression of GSDM^NT^. This strategy not only induces pyroptosis but also enhances anti-tumor responses. Notably, better therapeutic effects have been achieved when combined with anti-PD-L1 [170].

#### Potential strategies targeting GSDMA

Bioorthogonal chemistry is a novel technology for observing biological processes including cell death and immunity. Wang et al. established a bioorthogonal chemical system in which the cancer-imaging probe phenylalanine trifluoroborate (Phe-BF3) can selectively cleave GSDMA3, which is linked to a nanoparticle by conjugate [121]. After injection with this bioorthogonal system, Hela, EMT6, and 4T1 cells induced pyroptosis initiation and augmented antitumor immune responses, but not in immune-deficient mice.

#### Potential strategies targeting GSDMB

Traditionally, researchers have believed that cytotoxic lymphocyte-derived granzyme-mediated cell death primarily leads to apoptosis. However, Zhou et al. found that NK cells and CTL-derived GZMA can cleave GSDMB, inducing the initiation of pyroptosis [20]. IFN-γ has also been identified as a regulator that upregulates GSDMB expression and facilitates GZMA-mediated pyroptosis. Thus, cytotoxic lymphocytes may provide a new strategy for antitumor therapy by inducing gasdermin-mediated pyroptosis.

As GSDMB is highly expressed in most HER2 breast cancers and leads to resistance to anti-HER2 treatments, researchers have developed a nanocapsule loaded with an anti-GSDMB antibody and targeted GSDMB-overexpressing cancer cells. Furthermore, it is associated with increased tumor cell apoptosis, decreased tumor progression, and the elimination of anti-HER2 drug resistance [171].

#### Potential strategies targeting GSDMC

In recent years, cancer immunotherapy using bacteria has proven highly successful in augmenting immune responses and impeding tumor progression [172]. A notable discovery by Liu et al. unveiled that intravenous administration of living Listeria not only recruits immune cells, triggering an inflammatory response with antitumor effects, but also elevates the expression of GSDMC and caspase-8, thereby promoting tumor pyroptosis. This orchestrated process involves the release of inflammatory factors, such as IL-18 and IL-1β, which attract dendritic cells and CD8^+^ T cells into the tumor tissue, activating antitumor immunity and hindering tumor progression [173]. Consequently, the integration of bacteria-based immunotherapy with pyroptosis has emerged as a promising strategy for antitumor treatment. As detailed earlier, PD-L1facilitates the transition from TNF-α-induced apoptosis to pyroptosis by activating and cleaving GSDMC [68]. While nearly all chemotherapy drugs can enhance GSDMC expression and promote nPD-L1 translocation, specific antibiotics, such as daunorubicin, doxorubicin, epirubicin, and actinomycin-D, have been shown to activate caspase-8 and then cleave GSDMC, initiating pyroptosis in cancer cells. Therefore, these drugs present a promising and innovative anti-tumor strategy [68].

#### Potential strategies targeting GSDMD

As a neoadjuvant chemotherapy agent, cisplatin has demonstrated efficacy in elevating cure rates and inducing a pathological complete response in triple-negative breast cancer [174]. While some studies have attributed the cytotoxic effects of chemotherapeutic drugs to the induction of apoptosis, the precise mechanism by which cisplatin operates in breast cancer necessitates further exploration [175]. Yanet al. found that cisplatin induces pyroptosis in breast cancer cells by activating the caspase-1/GSDMD pathway and then upregulating the expression of the long non-coding RNA maternally expressed gene 3 (MEG3). Notably, the antitumor effects induced by cisplatin were found to be inhibited upon blocking MEG3 [175]. This finding may provide a novel therapeutic strategy for the treatment of triple-negative breast cancer [174].

Cucurbitacin B, a natural triterpenoid derived from the Cucurbitaceae plant, has been extensively studied for its involvement in the apoptosis pathway across various cancer types [176–178]. A recent study found that cucurbitacin B could also induce GSDMD-mediated pyroptosis in NSCLC by binding to TLR4 and increasing ROS induction and cytosolic calcium release. Cucurbitacin B, in particular, not only inhibits tumor progression through pyroptosis, but also protects normal organ tissues [179]. Therefore, cucurbitacin B is a promising therapeutic agent for treating NSCLC.

Ding et al. first synthesized the biodegradable K3ZrF7: Yb/Er upconversion nanoparticles (ZrNPs) as pyroptosis inducers, which can be dissolved into cancer cells and induce GSDMD cleavage, caspase-1 activation, and IL-1β release [180]. Furthermore, in vivo animal experiments confirmed that this nanoparticle can also enhance antitumor immunity by increasing dendritic cell maturity and effector-memory T-cell infiltration. These nanoparticles will provide a theoretical basis for clinical applications. Subsequently, a multivalent CXCR4-targeted nanotoxin was developed to induce GSDMD-mediated pyroptosis and inhibit colorectal cancer progression [181]. These nanoparticles can target colorectal cancer cells through interactions with the CXCR4 receptor, which is overexpressed on the surface of colorectal cancer stem cells, and effectively overcome chemoresistance by triggering pyroptosis rather than apoptosis.

GSDMD activates the cGAS pathway via K^+^ efflux and promotes PD-L1 expression via the Ca^2+^/HDACs/STAT1 signaling pathway in human hepatocellular carcinoma. Elevated levels of GSDMD expression positively correlated with PD-L1 expression and predicted poor prognosis in patients with hepatocellular carcinoma. Combined treatment with anti-PD-L1 and GSDMD inhibitors enhances antitumor effects and inhibits metastasis of hepatocellular carcinoma cells [103].

As enhancer deregulation has been demonstrated to be associated with oncogenesis, Ning and colleagues found that deletion of mixed-lineage leukemia 4 (MLL4), an enhancer-associated histone H3 lysine 4 mono-methyltransferase, has the potential to heighten anti-tumor immunity by inducing GSDMD-mediated pyroptosis and transcriptional reactivation of the double-stranded RNA-interferon response. GSDMD-mediated pyroptosis can enhance the efficacy of anti-PD-1 therapy in melanomas [182].

#### Potential strategies targeting GSDME

Recent studies have revealed that chemotherapy can induce GSDME-mediated pyroptosis by cleaving caspase-3. For instance, decitabine has been shown to upregulate GSDME expression, shifting apoptosis to pyroptosis in tumor cells [67], and pretreatment with decitabine has augmented the effectiveness of nanodrug-delivered cisplatin in triple-negative breast cancer [183]. In melanoma, a combination of a BRAF inhibitor and a MEK inhibitor-induced GSDME-mediated pyroptosis, contributes to increased antitumor immunity [184]. Triptolide, a natural diterpene epoxide with potent antitumor activity, triggers GSDME-mediated pyroptosis in head and neck cancers [26]. Triptolide inhibits the expression of c-Myc and mitochondrial hexokinase II, leading to caspase-3 activation and subsequent cleavage by active caspase-3. Similarly, other chemotherapeutics, such as lobaplatin, doxorubicin, and paclitaxel, can induce cancer cells to undergo pyroptosis, rather than necroptosis, by activating caspase-3 to cleave GSDME in colorectal and lung cancer [185, 186]. These studies illustrate that the therapeutic effect of chemotherapeutic drugs can be strengthened by converting apoptosis to pyroptosis.

Immunotherapy has been shown to function as a tumor suppressor in various tumors such as melanoma [187], non-small cell lung cancer [188], and bladder cancer [189]. However, “cold” tumors have a poor response to immunotherapy, including prostate cancer [190]. Therefore, it is important to explore effective strategies for the treatment of these cancers. Wu et al. found that inhibition of CDC20 can significantly enhance the anti-tumor immunity of prostate through activating GSDME-mediating pyroptosis [191]. Thus, targeting pyroptosis offers a potential strategy for the therapy of “cold” cancer.

Recently, nanotechnology and nanomaterials have been more widely used in clinical practice. A carrier-free nanoplatform assembled with cytarabine and chlorin e6 was designed to induce pyroptosis in breast cancer [192]. This nanoplatform specifically targets GSDME-mediated pyroptosis by accumulating ROS and inducing immunogenic cell death. Resulting in the release of HMGB1, ATP, and calcitonin, ultimately leading to GSDME activation and cleavage. Additionally, this nanoplatform has demonstrated the ability to stimulate cytotoxic T lymphocyte maturation, making it a valuable therapeutic strategy for targeting pyroptosis and enhancing the antitumor immune response [192]. Zhao et al. introduced a biomimetic nanoparticle (BNP) comprising a poly (lactic-co-glycolic acid) polymeric core and cancer cell membrane cloak. Loaded with indocyanine green and decitabine, this nanoparticle effectively induced cancer cell pyroptosis and activated an antitumor immune response, thereby inhibiting tumor progression and metastasis [193]. It has been confirmed that patients with overexpression of CXCR4 have a worse prognosis for head and neck squamous cell carcinoma; thus, it is urgent and necessary to find new therapeutic approaches targeting CXCR4 [194, 195]. Blanco et al. designed nanotoxins with CXCR4-dependent cytotoxic effects. Notably, these nanotoxins can also activate caspase-3 and induce GSDME-mediated pyroptosis, which may activate immune cells and boost antitumor immunity [196]. To enhance antitumor efficacy and minimize side effects, researchers designed a nanotechnology platform that can induce tunable cellular pyroptosis with up to 40-fold tunability in GSDME-expressing cancers [197]. This study provides new insights for exploring nanomaterial-mediated pyroptosis as a strategy for cancer treatment.

As a novel treatment modality, oncolytic viruses (OV) encompass native or engineered viruses with the ability to selectively target tumor cells and facilitate antitumor immunity [198–200]. Metformin, an antidiabetic drug, has been confirmed to play a critical role in anti-tumor responses [201]. However, the specific underlying mechanism remained unclear. Recent findings indicate that metformin activates AMPK/SIRT1 signaling, promotes NF-κB expression, and induces caspase-3/GSDME-mediated pyroptosis in various cancer cells, including hepatoblastoma, colon cancer, and breast cancer cells [202]. Therefore, pyroptosis induced by metformin may be a potential therapeutic target for the treatment of multiple tumors. In triple-negative breast cancer (TNBC), GSDME is activated along with the overexpression of mitochondrial uncoupling protein 1 (UCP1). Overexpression of UCP1 promotes mitochondrial destruction and pyroptosis, and inhibits TNBC proliferation and metastasis.

### Potential strategies targeting pyroptosis-related inflammatory cytokines

Inflammatory cytokines released during pyroptosis are crucial regulators of tumor progression and metastasis. Therefore, targeting inflammatory cytokines may provide potential opportunities for the treatment of various cancers. Canakinumab, a human anti-IL-1β monoclonal antibody extensively used in inflammatory diseases, has been used in the treatment of various cancers, including lung cancer [203, 204], breast cancer [205], colon cancer, and other tumors [206].Furthermore, Yuan et al. demonstrated that the inhibition of IL-1β with canakinumab significantly reduced tumor growth in K-ras-mutant lung adenocarcinoma by reshaping the TME [133]. The anti-IL-1β mAb promoted the infiltration and activation of CD8^+^ T cells while suppressing the function of myeloid-derived suppressor cells. This effect was also observed in the 4T1 mouse model, where anti- IL-1β Abs significantly enhanced the antitumor effect of anti-PD-1 Abs [128]. Therefore, blocking IL-1β may be a promising therapeutic strategy for K-ras–mutant lung adenocarcinoma. Similarly, inhibiting the IL-1β pathway with an IL-1 receptor antagonist (IL-1Ra) may play a crucial role in suppressing tumor growth [207]. Anakinra, an IL-1Ra, has been shown to suppress breast cancer growth by reducing the secretion of IL-1β and IL-22 [208]. Additionally, anakinra has proven effective in significantly mitigating cytokine release syndrome during CAR-T therapy, offering a promising strategy to address the severe side reactions associated with this therapy [209–211].

## Perspective and conclusion

In this review, we delved into the intricate molecular mechanisms of pyroptosis and explored its potential as a therapeutic strategy in cancer treatment. Pyroptosis, with its dual impact on tumor progression mainly through two approaches: On the one hand, the expression of pyroptosis-associated genes affects tumor progression and patient prognosis. On the other hand, pyroptosis can influence anti-tumor immune responses. The induction of pyroptosis enhances immune activity by upregulating CD8^+^ T cells, NK cells, and M1 macrophage infiltration [121]. Therefore, combining pyroptosis-based therapies with immunotherapy may be a promising treatment for tumors.

Despite significant advancements, pyroptosis-mediated therapy still faces significant challenges. First, studies have established that chemotherapy can induce apoptosis and transition to pyroptosis by cleaving gasdermins, which may be a promising approach [67]. Gasdermins are expressed at low levels in some tumors but are highly expressed in normal cells [69, 86, 105]. Future research should focus on developing strategies to restore gasdermin expression in tumor cells and devise specifically targeted agonists to mitigate the potential side effects. Second, although pyroptosis has been confirmed to play a crucial role in antitumor immunity, researchers have observed associations between pyroptosis induction and tumor progression with poor prognosis in certain cancers. For instance, overexpression of GSDMC has been linked to a worse prognosis in lung adenocarcinoma [96], breast cancer [68], and gastric cancer [97]. Moreover, the activation of caspase-8 by PD-L1, leading to GSDMC-mediated pyroptosis induction, has been implicated in promoting tumor development and suppressing the antitumor immune response [68]. Therefore, comprehensive studies are imperative to elucide the specific role of pyroptosis in malignant tumors. Third, the release of inflammatory cytokines during pyroptosis induction has been shown to reprogram the TME and restore antitumor immunity. However, pyroptosis-derived pro-inflammatory cytokines may induce severe CRS in CAR-T therapy, limiting their application in tumors [19]. Consequently, controlling the side effects induced by pyroptosis during CAR-T therapy warrants further investigation.

In summary, pyroptosis has emerged as a pivotal player in tumor development and progression, and offer novel targets for therapeutic intervention. The GSDM family is the principal executor of pyroptosis and presents itself as a promising avenue for effective therapeutic strategies in future treatments. Consequently, comprehensive and in-depth investigations are essential to unravel the intricate effects and molecular mechanisms underlying pyroptosis and GSDM family. These endeavors will pave the way for the development of innovative pyroptosis-associated treatment strategies.

## Data Availability

Not applicable.

## Abbreviations

IL-1β: Interleukin-1β
GSDMD: Gasdermin D
GSDM: Gasdermin
PJVK: Pejvakin
Rim3: Recombination-induced mutation 3
CAR-T: Chimeric antigen receptor T cell
GZMB: Granzyme B
CRS: Cytokine release syndrome
HMGB1: High-mobility group box protein 1
TNFR: Tumor necrosis factor receptors
TRAIL-R: TNF-related apoptosis-inducing ligand receptors
TLRs: Toll-like receptors
FADD: Fas-associated death domain protein
TRADD: TNFR1-associated death domain protein
DISC: Death-inducing signaling complex
APAF-1: Apoptotic activating factor 1
RIPK1: Receptor interacting protein kinase 1
MLKL: Mixed lineage kinase-like
PPRs: Pattern recognition receptors
PAMPs: Pathogen-associated molecular patterns
DAMPs: Damage-associated molecular patterns
NLRs: NOD-like receptors
CLRs: C-type lectin receptors
RIG-1: Retinoic acid-inducible gene 1
RLRs: RIG-1-like receptors
NLRP: NLR family pyrin domain-containing
AIM2: Absent in melanoma 2
ASC: Adaptor protein apoptosis-associated speck-like protein containing a caspase recruitment domain
CARD: Caspase activation and recruitment domain
LPS: Lipopolysaccharide
ESCRT: Endosomal sorting complexes required for transport
ATP: Adenosine triphosphate
P2X7R: P2X7 receptor
TNF-α: Tumor necrosis factor-alpha
TME: Tumor microenvironment
LMO1: LIM domain only 1
TGF-β: Transforming growth factor- beta
UP24: Ubiquitin-specific peptidase 24
STAT3: Signal transducer and activator of transcription 3
NK: Natural killer
CTLs: Cytotoxic T lymphocytes
MLZE: Melanoma-derived leucine zipper-containing extranuclear factor
α-KG: α-Ketoglutarate
TCA: Tricarboxylic acid
ROS: Reactive oxygen species
DR6: Death receptor 6
NSCLC: Non-small cell lung cancer
EGFR: Epidermal growth factor receptor
TCGA: The Cancer Genome Atlas
APCs: Antigen-presenting cells
ICERE-1: Inversely correlated with estrogen receptor expression
CpGs: Clinical practice guidelines
TNM: Tumor-node-metastasis
TAMs: Tumor-associated macrophages
ICD: Inflammatory cell death
MDSC: Myeloid-derived suppressor cell
CCL2: Chemokine (CC-motif) ligand 2
KRAS: Kirsten rat sarcoma viral oncogene homolog
EMT: Epithelial-mesenchymal transition
VEGF: Vascular endothelial growth factor
CXCL2: C-X-C motif chemokine ligand 2
IFN-γ: Interferon-gamma
IL-18BP: IL-18 binding protein
DR-18: Decoy-resistant IL-18
ERK1/2: Extracellular signal-related kinases 1 and 2
Tregs: Regulatory T lymphocytes
ECM: Extracellular matrix
MEG3: Maternally expressed gene 3
MLL4: Mixed-lineage leukemia 4
BNP: Biomimetic nanoparticle
OV: Oncolytic viruses
TNBC: Triple-negative breast cancer
UCP1: Uncoupling protein 1
IL-1Ra: IL-1 receptor antagonist
